# RNA-binding protein Rbm38 as a multifaceted post-transcriptional regulator in zebrafish pancreatic development

**DOI:** 10.1093/jmcb/mjaf025

**Published:** 2025-08-12

**Authors:** Xiangmin Zhang, Xianpeng Li, Rui Liu, Lu Wang, Yunchao Wang, Ailong Zhang, Shuaiqi Yang, Hongyan Li

**Affiliations:** College of Marine Life Sciences, Key Laboratory of Evolution & Marine Biodiversity (Ministry of Education) and Institute of Evolution & Marine Biodiversity, Ocean University of China, Qingdao 266003, China; College of Marine Life Sciences, Key Laboratory of Evolution & Marine Biodiversity (Ministry of Education) and Institute of Evolution & Marine Biodiversity, Ocean University of China, Qingdao 266003, China; College of Marine Life Sciences, Key Laboratory of Evolution & Marine Biodiversity (Ministry of Education) and Institute of Evolution & Marine Biodiversity, Ocean University of China, Qingdao 266003, China; College of Marine Life Sciences, Key Laboratory of Evolution & Marine Biodiversity (Ministry of Education) and Institute of Evolution & Marine Biodiversity, Ocean University of China, Qingdao 266003, China; College of Marine Life Sciences, Key Laboratory of Evolution & Marine Biodiversity (Ministry of Education) and Institute of Evolution & Marine Biodiversity, Ocean University of China, Qingdao 266003, China; College of Marine Life Sciences, Key Laboratory of Evolution & Marine Biodiversity (Ministry of Education) and Institute of Evolution & Marine Biodiversity, Ocean University of China, Qingdao 266003, China; College of Marine Life Sciences, Key Laboratory of Evolution & Marine Biodiversity (Ministry of Education) and Institute of Evolution & Marine Biodiversity, Ocean University of China, Qingdao 266003, China; College of Marine Life Sciences, Key Laboratory of Evolution & Marine Biodiversity (Ministry of Education) and Institute of Evolution & Marine Biodiversity, Ocean University of China, Qingdao 266003, China; Laboratory for Marine Biology and Biotechnology, Qingdao Marine Science and Technology Center, Qingdao 266003, China

**Keywords:** Rbm38, pancreas, post-transcriptional regulation, zebrafish

## Abstract

RNA-binding motif protein 38 (Rbm38), also known as RNPC1, is a major regulator of post-transcriptional gene expression. It represents a potential candidate gene linked to the susceptibility of type 2 diabetes, and decreased RBM38 expression can enhance the proliferation of pancreatic cancer cells in humans. However, its role in pancreatic development remains elusive. In this study, we explored the function of Rbm38 using zebrafish as a model. Pancreatic expression of Rbm38 is present at larval stages and is controlled by several transcription factors acting on specific *rbm38* promoter regions. The loss of Rbm38 leads to abnormal pancreatic enlargement. Mechanistically, Rbm38 is involved in several aspects of post-transcriptional regulation of pancreatic gene expression. It destabilizes *pdx1* transcripts by binding to the 3′-untranslated region and regulates alternative splicing of key pancreatic transcription factor genes, including *isl1a, smad2*, and *nkx2.2a*. These findings elucidate the role of Rbm38 in pancreatic development and highlight its significance in maintaining pancreatic homeostasis.

## Introduction

The development and maintenance of pancreatic homeostasis are governed by a complex interplay of regulatory mechanisms, encompassing both transcriptional and post-transcriptional controls (Apelqvist et al., 1999; Hebrok, 2003; Fujitani et al., 2006; Glauser and Schlegel, 2009; Leung, 2010; Jennings et al., 2015; Mi et al., 2024). Although previous works mostly centered on transcriptional regulation, there is a growing body of evidence indicating that post-transcriptional regulation is equally vital for proper pancreatic development. RNA-binding proteins (RBPs) are key players of post-transcriptional regulation, harboring one or more RNA recognition motifs (RRMs) that allow them to regulate RNA metabolism at multiple levels, from pre-RNA splicing to mRNA localization, polyadenylation, stability, translation, and nuclear export (Li et al., 2010). These multifaceted RBPs are integral to a diverse array of biological processes, highlighting their importance in the intricate regulation of pancreatic function (Blencowe, 2006; Grifone et al., 2020).

Several RBPs have been identified as crucial players in pancreatic development and homeostasis. Recent studies using various animal models have elucidated multiple post-transcriptional regulatory mechanisms by which they function within the pancreas. For instance, PTBP binds to the pyrimidine-rich region in the 3′-untranslated region (3′-UTR) of rat *insulin* mRNA, promoting its stability and translation (Tillmar et al., 2002). The translocation of PTBP1 between the nucleus and cytoplasm is also linked to the rapid post-transcriptional regulation of insulin levels in pancreatic islets, which are involved in the response to glucose levels. This regulatory process may be disrupted in type 2 diabetes, leading to impaired insulin secretion (Ehehalt et al., 2010). Furthermore, RBPs are involved in translation control, which is another essential aspect of pancreatic homeostasis. RBMS3, for example, facilitates the translation of *Ptf1a* mRNA by interacting with its 3′-UTR (Lu et al., 2012). HuD serves as a key regulator of *insulin* mRNA translation in pancreatic β-cells by binding to the 5′-UTR of pre-proinsulin (*ins2*) mRNA, inhibiting its translation, and consequently decreasing insulin production. Alternative splicing represents another layer of gene regulation, contributing to pancreatic development. Rbm4 modulates pancreatic cell differentiation and insulin secretion by coordinating the alternative splicing of key genes encoding pancreatic developmental transcription factors, such as Isl1 and Pax4 (Lin et al., 2013). NOVA1 plays an important role in the functionality of pancreatic β-cells, influencing insulin secretion and apoptosis by regulating alternative splicing events (Villate et al., 2014). Despite the progress made in understanding the post-transcriptional regulation of pancreatic development, there remains an urgent need for identifying additional RBPs that participate in this process in order to gain further insight into the molecular underpinnings of pancreatic function and disease.

Rbm38 contains a single RRM domain and is involved in various post-transcriptional regulatory processes. It binds to AU-rich or GU-rich regions in the 3′-UTR of target mRNAs, regulating mRNA metabolism through multiple mechanisms, including stabilization, destabilization, and/or translation (Shu et al., 2006; Zhang et al., 2010; Cho et al., 2012; Yan et al., 2012; Xu et al., 2013; Ye et al., 2018; She et al., 2020). For instance, Rbm38 stabilizes *p53, p21*, and *p73* transcripts, thereby promoting their translation (Shu et al., 2006; Zhang et al., 2011; Yan et al., 2012). Conversely, it promotes the degradation of mRNAs encoding oncogenic proteins such as MYC and MDM2 (Xu et al., 2013; Li et al., 2017). Additionally, Rbm38 inhibits the translation of *p53* mRNA by preventing eIF4E from binding to its 5′ cap region (Zhang et al., 2011). Furthermore, Rbm38 acts as a tissue-specific splicing factor, regulating the splicing of numerous genes involved in erythroid differentiation, antiviral immunity, neural differentiation, and muscle development (Anyanful et al., 2004; Heinicke et al., 2013; Du et al., 2020; Li et al., 2020). Previous studies suggested that Rbm38 is a candidate gene for the susceptibility to type 2 diabetes, and the decreased expression of Rbm38 may promote the proliferation of pancreatic cancer cells (Saxena et al., 2013; Gong et al., 2016; Lu et al., 2022). However, no causal relationship between Rbm38 and pancreatic development has been established to date.

Here, we uncover a novel function of Rbm38 in zebrafish pancreatic development. Rbm38 displays prominent expression in the pancreas at larval stages. The deficiency of Rbm38 leads to abnormal enlargement of the pancreas during early development. Mechanistically, Rbm38 regulates pancreatic development by destabilizing *pdx1* transcripts and controlling the alternative splicing of key genes encoding pancreatic transcription factors, including *isl1a, smad2*, and *nkx2.2a*. These data demonstrate a critical role of Rbm38 in zebrafish pancreatic development by post-transcriptionally controlling the expression of pancreatic genes.

## Results

### Zebrafish Rbm38 shows restricted expression in the developing pancreas

**Figure 1:**
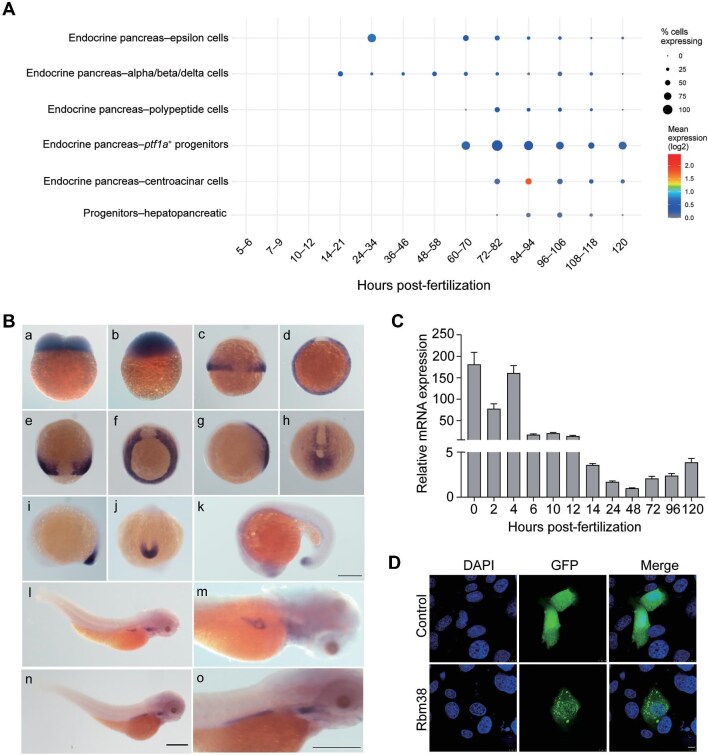
Expression of *rbm38* during zebrafish early development. (**A**) Expression pattern of zebrafish *rbm38* in endoderm cell lineages. The analysis was performed using online single-cell transcriptome database (https://daniocell.nichd.nih.gov/index.html). The size of each dot reflects the relative mean expression level of the corresponding gene. (**B**) WISH analysis of zebrafish *rbm38* expression at different developmental stages, including 2-cell stage (a), 4 hpf (b), 6 hpf (c, lateral view; d, animal pole view), 9 hpf (e, dorsal view; f, vegetal pole view), 10 hpf (g, lateral view; h, posterior view), 12 hpf (i, lateral view; j, posterior view), 18 hpf (k), 72 hpf (l; m, higher magnification), and 96 hpf (n; o, higher magnification). Scale bar, 300 μm. (**C**) RT-qPCR analysis of *rbm38* temporal expression levels during early development. Data are normalized to *gapdh* and presented as mean ± SD. (**D**) Subcellular localization of Rbm38-GFP in SW1990 cells. Nuclei are counterstained by DAPI. Scale bar, 10 μm.

Gene function is often closely related to its expression pattern. The expression profile of *rbm38* in the online single-cell transcriptome database (Sur et al., 2023) indicates that it is expressed in endocrine and exocrine cells as well as hepato–pancreatic progenitor cells, with significantly higher expression levels in exocrine cells than in endocrine cells (Figure 1A).

To analyze the expression pattern of *rbm38* during early stages of zebrafish development, we conducted whole-mount *in situ* hybridization (WISH) using an antisense RNA probe (Figure 1B). The expression of *rbm38* was observed from the 2-cell stage to 96 h post-fertilization (hpf). Prior to the onset of zygotic gene expression, *rbm38* displayed a robust ubiquitous expression pattern (Figure 1B, a and b), indicating that it is a maternal gene. From 6 hpf to 9 hpf, *rbm38* was specifically expressed in the mesoderm except for the dorsal region representing the precursor of the notochord (Figure 1B, c–f). From 10 hpf to 24 hpf, *rbm38* was specifically expressed in the tail bud, eyes, and unsegmented presomitic mesoderm (Figure 1B, g–k). Between 72 hpf and 96 hpf, *rbm38* was primarily detected in the pharyngeal arches and pancreas (Figure 1B, l–o). Quantitative real-time PCR (RT-qPCR) analysis revealed that the temporal expression of *rbm38* exhibited a biphasic pattern, with high levels at initial stages of development, progressively decreasing to the lowest level at 48 hpf, and then rising again (Figure 1C).

We next examined the subcellular localization of Rbm38 by expressing GFP or Rbm38-GFP fusion protein in SW1990 cells. The results revealed that GFP was expressed uniformly in the cell, while Rbm38-GFP was widely distributed in both the cytoplasm and the nucleus, displaying a granular pattern in the cytoplasm (Figure 1D). This suggests that Rbm38, as an RBP, may exert function through the formation of ribonucleoprotein (RNP) granules.

**Figure 2:**
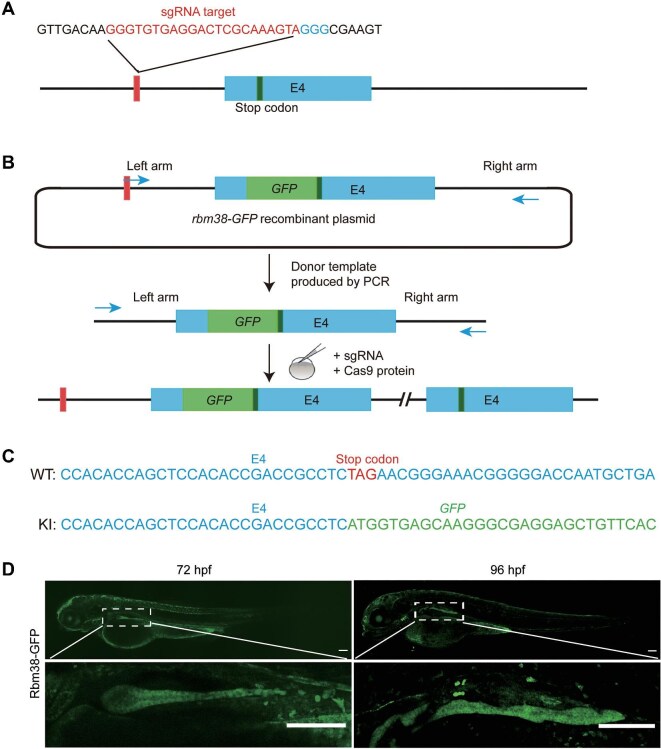
Generation of zebrafish *rbm38*-*GFP* knock-in line by CRISPR/Cas9. (**A**) Schematic of the intron targeting-mediated strategy for generating *GFP* knock-in at the zebrafish *rbm38* locus. The sgRNA target sequence is shown in red and the protospacer adjacent motif (PAM) sequence in blue. (**B**) The *GFP* coding sequence was integrated into the *rbm38* locus after co-injection of the donor template with the sgRNA and Cas9 protein. The left arm is 1497 bp, and the right arm is 2120 bp. (**C**) Genotyping of the *rbm38*-*GFP* knock-in line. Blue indicates the *rbm38* exon 4 sequence, green shows the *GFP* sequence, and red marks the *rbm38* stop codon. (**D**) Expression of endogenous Rbm38 at 72 hpf and 96 hpf. Lower panels are higher magnifications of the insets. Scale bar, 100 μm.

Zebrafish Rbm38-specific antibody is not available. To examine the expression pattern of endogenous Rbm38 protein, we set out to generate an *rbm38-GFP* knock-in zebrafish line. A linear donor template comprised of the last exon and partial flanking sequences fused with the *GFP* coding sequence was co-injected with Cas9 protein and a specific small guide RNA (sgRNA) into 1-cell-stage embryos (Figure 2A–C). In the offspring, the expression pattern of Rbm38 protein was consistent with the data obtained by WISH. Notably, a robust expression was observed in the pancreas at 72 hpf and 96 hpf (Figure 2D). The pancreatic localization of Rbm38-GFP was further confirmed by co-staining using a pancreas-specific antibody for carboxypeptidase A (Supplementary Figure S1).

**Figure 3:**
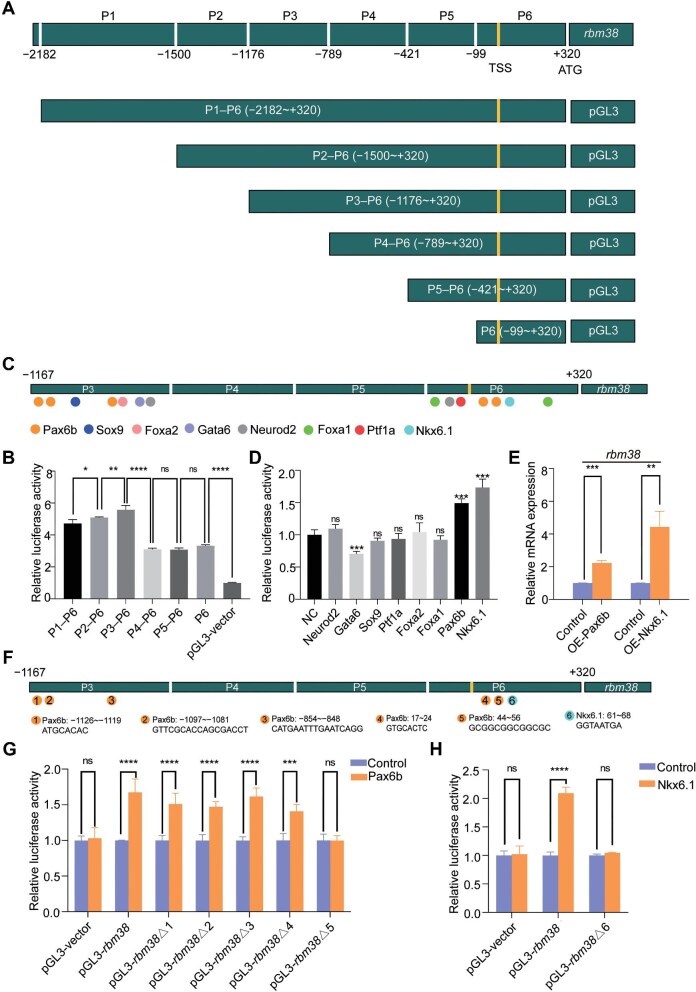
Regulation of *rbm38* expression by Pax6b and Nkx6.1. (**A**) Schematic diagram illustrating reporter constructs with different *rbm38* promoter regions (P1–P6). (**B**) The *rbm38* promoter-driven luciferase activity in HEK293T cells. The value from cells transfected with pGL3 vector was set to 1, and data were normalized to Renilla luciferase activity. (**C**) Mapping of putative pancreatic developmental transcription factor-binding sites within the P3 and P6 regions of the *rbm38* promoter. (**D**) Regulation of *rbm38* promoter (P3–P6) activity by different transcription factors in HEK293T cells. The value from cells transfected with pcDNA3.1 empty vector (NC) was set to 1, and data were normalized to Renilla luciferase activity. (**E**) Quantitative analysis of *rbm38* expression in embryos overexpressing (OE) Pax6b and Nkx6.1 at 72 hpf. (**F**) Mapping of the putative Pax6b- or Nkx6.1-binding sites in the P3 and P6 regions of the *rbm38* promoter. (**G** and **H**) Binding site 5 (GCGGCGGCGGCGC) and binding site 6 (GGTAATGA) are essential for Pax6b and Nkx6.1 to regulate luciferase activity in HEK293T cells, respectively. Values in pcDNA3.1 empty vector-transfected cells were set to 1 after normalization to Renilla luciferase activity. Data are presented as mean ± SD (one-way ANOVA; *P < 0.05; **P < 0.01; ***P < 0.001; ****P < 0.0001; ns, not significant).

To understand the mechanism underlying *rbm38* expression in the pancreas, we examined its transcriptional regulation. The full-length *rbm38* promoter and its sequential deletions (P1–P6, P2–P6, P3–P6, P4–P6, P5–P6, and P6) were cloned into the pGL3 vector (Figure 3A) and transfected into HEK293T cells. Reporter gene assays indicated that two of the six regions (P3 and P6) are likely responsible for regulating *rbm38* gene expression (Figure 3B). We then searched for possible pancreas-related transcription factors that might bind to promoter segments P3 and P6 using online tools and identified eight putative pancreatic developmental transcription factors, including Neurod2, Gata6, Sox9, Ptf1a, Foxa1, Foxa2, Pax6b, and Nkx6.1 (Figure 3C). To examine whether they bind to P3 and P6 regions, their coding regions were individually cloned into the pcDNA3.1 vector and co-transfected with pGL3-(P3–P6) into HEK293T cells (Supplementary Figure S2). The dual-luciferase assay revealed that Pax6b and Nkx6.1 could activate *rbm38* expression, whereas Gata6 seemed to have an inhibitory effect (Figure 3D). Consistently, injection of *pax6b* or *nkx6.1* mRNA into 1-cell-stage zebrafish embryos led to a significant upregulation of *rbm38* expression (Figure 3E).

Subsequently, luciferase reporter constructs with deletions of Pax6b- or Nkx6.1-binding sites were generated (pGL3-*rbm38*Δ1–pGL3-*rbm38*Δ6) (Figure 3F). The results showed that the transcriptional activity of the *rbm38* promoter significantly decreased after the deletion of candidate binding site 5 (GCGGCGGCGGCGC) for Pax6b and candidate binding site 6 (GGTAATGA) for Nkx6.1, indicating that these sites may play a crucial role in activating *rbm38* expression (Figure 3G and H).

### Generation of rbm38 mutants

**Figure 4:**
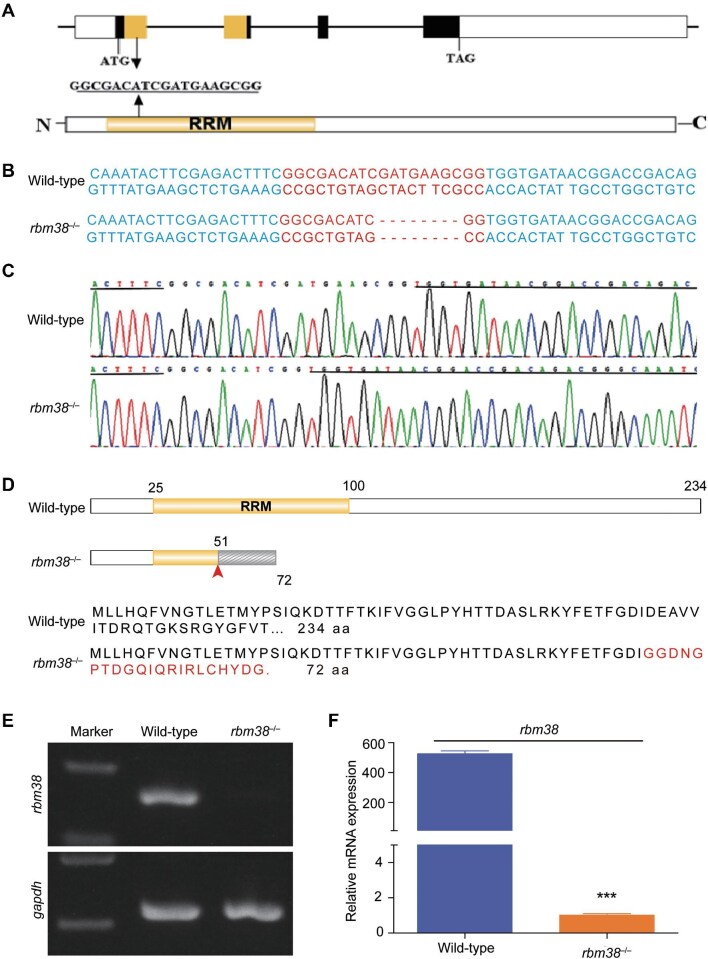
Generation of zebrafish *rbm38* mutant line by CRISPR/Cas9-mediated genome editing. (**A**) Location of the sgRNA target site in the zebrafish *rbm38* locus. (**B** and **C**) Genotyping of *rbm38* mutation. The sgRNA target sequence is highlighted in red, and the indel is indicated by dashes. (**D**) Protein domain and amino acid sequence of wild-type and mutant Rbm38. Residues translated after a frameshift are shown in red. (**E** and **F**) PCR and RT-qPCR analyses of *rbm38* expression levels in wild-type and *rbm38* mutant embryos at 72 hpf, with *gapdh* as a control. Data are presented as mean ± SD (Student's *t*-test; ***P < 0.001).

To analyze the function of Rbm38 in pancreatic development, we set out to create a zebrafish *rbm38* mutant line using CRISPR/Cas9 technology by targeting the first exon, which encodes the RRM domain (Figure 4A). An 8-bp deletion was obtained, and the mutation is expected to cause a premature termination of protein translation, resulting in a truncated peptide with only 72 amino acids (Figure 4B–D). Compared to the wild-type *rbm38*, the level of mutant *rbm38* mRNA was dramatically reduced (Figure 4E and F), likely due to nonsense-mediated RNA decay. No upregulated expression of the homologous genes *rbm24a* and *rbm24b* was observed in *rbm38* mutants (Supplementary Figure S3). In addition, *rbm38* maternal-zygotic mutants could develop into viable adults.

### Deficiency of Rbm38 causes abnormal enlargement of the pancreas

**Figure 5:**
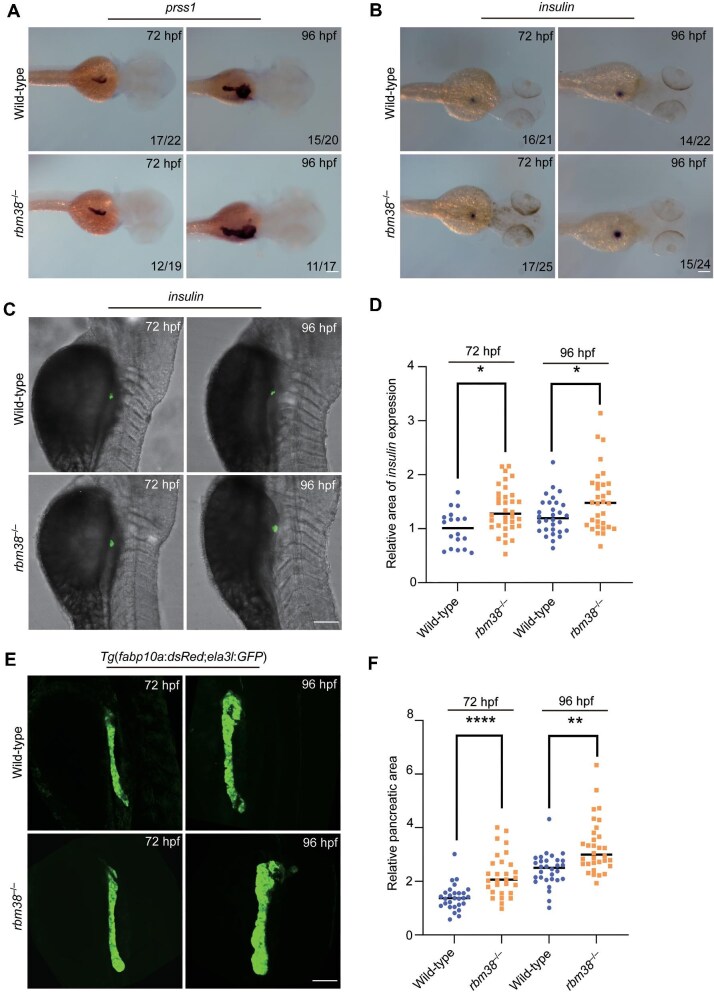
Loss of Rbm38 leads to pancreas enlargement. (**A** and **B**) WISH analysis of pancreatic marker gene expression in wild-type embryos and *rbm38* mutants at 72 hpf and 96 hpf. Lateral view. Scale bar, 100 μm. (**C**) FISH analysis of *insulin* expression in wild-type embryos and *rbm38* mutants at 72 hpf and 96 hpf. Lateral view. Scale bar, 100 μm. (**D**) Dot plot compares the area of *insulin* expression in wild-type embryos and *rbm38* mutants at 72 hpf. (**E**) Confocal z-stacks showing pancreas formation in wild-type embryos and *rbm38* mutants under *Tg*(*fabp10a*:*dsRed;ela3l*:*GFP*) background at 72 hpf and 96 hpf. Lateral view. Scale bar, 100 μm. (**F**) Dot plot compares the pancreatic area in wild-type embryos and *rbm38* mutants at 72 hpf. Data are presented as mean ± SD (Student's *t*-test; *P < 0.05; **P < 0.01; ****P < 0.0001).

To determine whether the deficiency of Rbm38 impacts pancreatic development, we first monitored the expression of the pancreatic endocrine marker gene *insulin* and the exocrine marker gene *prss1* at 72 hpf and 96 hpf using WISH. Both genes exhibited significantly expanded expression areas in the mutants compared to wild-type embryos at these stages (Figure 5A and B). Furthermore, fluorescence *in situ* hybridization (FISH) and quantitative analysis of the fluorescent signal area revealed a significant expansion of the *insulin* expression domain in *rbm38* mutants (Figure 5C and D). We then crossed *rbm38* mutants with the transgenic line *Tg*(*fabp10a*:*dsRed;ela3l*:*GFP*), which labels the liver and exocrine pancreas in red and green, respectively (Korzh et al., 2008). Consistent with the WISH results, the mutants displayed abnormal pancreatic enlargement at 72 hpf and 96 hpf (Figure 5E). Quantitative analysis of fluorescence areas at 72 hpf showed that the pancreas in *rbm38* mutants was significantly larger than that of sibling controls (Figure 5F).

**Figure 6:**
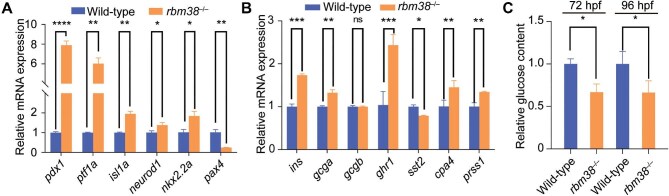
Loss of Rbm38 leads to pancreatic dysfunction. (**A**) Relative expression levels of *pdx1, ptf1a, isl1a, neurod1, nkx2.2a*, and *pax4* in wild-type embryos and *rbm38* mutants at 72 hpf. The expression level in wild-type embryos was set as 1 after normalization to *gapdh*. (**B**) Quantitative analysis of the expression levels of endocrine cell-related genes (*insulin, glucagon a, glucagon b, ghrelin, somatostatin*, and *sst2*) and exocrine cell-related marker genes (*cpa4* and *prss1*) in wild-type embryos and *rbm38* mutants at 72 hpf. The expression level in wild-type embryos was set as 1 after normalization to *gapdh*. (**C**) Relative glucose levels in wild-type and *rbm38* mutant tissues at 72 hpf and 96 hpf, with glucose level in wild-type embryos normalized to 1. Data are presented as mean ± SD (Student's *t*-test; *P < 0.05; **P < 0.01; ***P < 0.001; ****P < 0.0001; ns, not significant ).

We next examined the expression of transcription factor genes related to pancreatic development, including *pdx1, ptf1a, isl1a, pax4, nkx2.2a*, and *neurod1* at 72 hpf. Except for a distinctive downregulation of *pax4*, expression levels of the other genes were all upregulated, particularly for *pdx1* and *ptf1a* (Figure 6A). This upregulation was also observed at 96 hpf (Supplementary Figure S4A). We also analyzed the expression of endocrine cell-related genes *insulin, glucagon a, glucagon b, ghrelin*, and *somatostatin*, as well as exocrine cell-related marker genes *cpa4* and *prss1*. Compared with wild-type embryos, the expression of several pancreatic marker genes was dysregulated in *rbm38* mutants. Except for *glucagon b*, which showed no significant change, the expression level of *somatostatin* was downregulated, whereas expression levels of the other genes, particularly *insulin* and *ghrelin*, were significantly upregulated (Figure 6B; Supplementary Figure S4B). Additionally, glucose levels were significantly reduced in mutant embryos at 72 hpf and 96 hpf compared to wild-type embryos (Figure 6C). These findings suggest that the loss of Rbm38 disrupts the normal expression patterns of key pancreatic genes, which may cause the abnormal enlargement of the pancreas and potentially affect its function.

### Rbm38 binds to the 3′-UTR of pdx1 mRNA and reduces its stability

**Figure 7:**
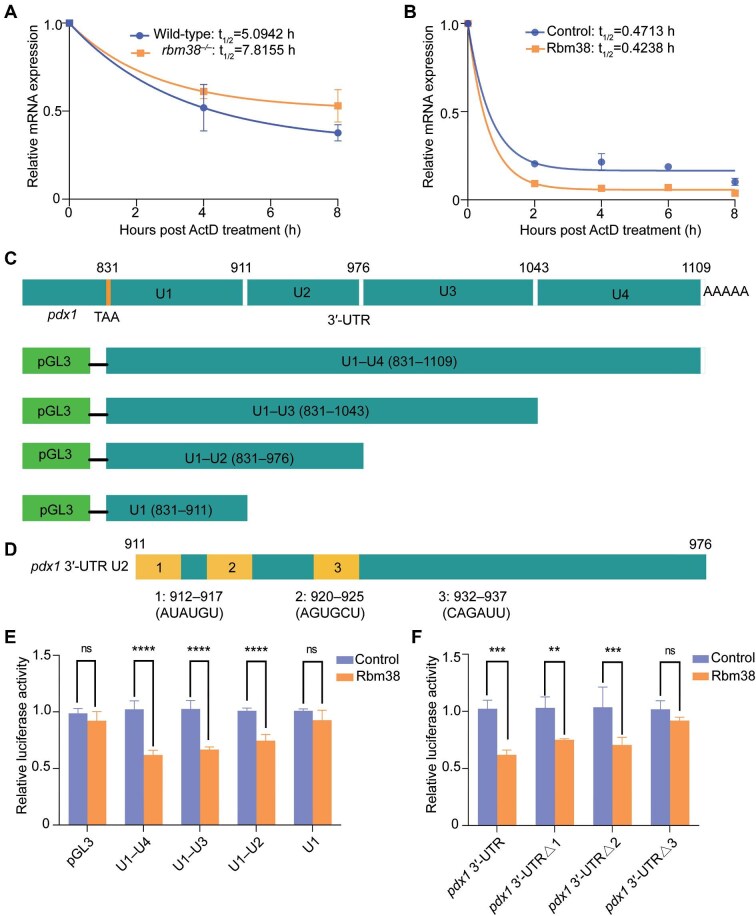
Rbm38 reduces the stability of *pdx1* mRNA by binding to its 3′-UTR. (**A**) The stability of *pdx1* mRNA after actinomycin D treatment in wild-type embryos and *rbm38* mutants at 72 hpf. (**B**) The stability of *pdx1* mRNA over time following actinomycin D treatment in control and HEK293T cells overexpressing Rbm38. (**C**) Schematic diagram illustrating the four segments (U1–U4) of zebrafish *pdx1* 3′-UTR fused with luciferase sequence. (**D**) Mapping of the three putative Rbm38-binding sites in the U2 region of *pdx1* 3′-UTR. (**E** and **F**) The CAGAUU sequence is required for Rbm38 to regulate luciferase activity in HEK293T cells. Values in empty vector transfected cells were set to 1 after normalization to Renilla luciferase activity. Data are presented as mean ± SD (One-way ANOVA; **P < 0.01; ***P < 0.001; ****P < 0.0001; ns, not significant).

Previous studies have shown that Rbm38 can regulate the stability of target mRNAs by binding to the 3′-UTR region (Zhang et al., 2010; Zou et al., 2021). To investigate the molecular mechanism underlying the elevated expression of key pancreatic developmental transcription factor genes *pdx1* and *ptf1a* in *rbm38* mutants, the stability of *pdx1* and *ptf1a* mRNAs was analyzed following actinomycin D treatment. We found that the half-life of *pdx1* mRNA in *rbm38* mutants was significantly longer than that in wild-type embryos (Figure 7A). However, the half-life of *ptf1a* showed no significant differences (Supplementary Figure S5A), suggesting that Rbm38 may regulate its expression through other mechanisms. Conversely, overexpression of Rbm38 in HEK293T cells reduced the stability of *pdx1* mRNA and accelerated its degradation (Figure 7B).

The full-length 3′-UTR of *pdx1* mRNA and different truncated forms were cloned into the pGL3 vector to generate luciferase reporter constructs (Figure 7C). These plasmids were then co-transfected with pcDNA3.1 or pcDNA3.1-*rbm38* into HEK293T cells. The results showed that Rbm38 inhibited the activity of luciferase reporters containing the U2 region (Figure 7E), suggesting that it may bind to this region within the *pdx1* 3′-UTR. In addition, we identified three potential Rbm38-binding sites within the U2 region using the online prediction tool and constructed luciferase reporters with deletions of these sites (*pdx1* 3′-UTRΔ1, *pdx1* 3′-UTRΔ2, and *pdx1* 3′-UTRΔ3) (Figure 7D). In contrast to the deletion of binding site 1 (AUAUGU) and binding site 2 (AGUGCU), the deletion of binding site 3 (CAGAUU) abolished the inhibitory effect of Rbm38 on luciferase activity (Figure 7F), suggesting that Rbm38 can bind to the CAGAUU sequence in the *pdx1* 3′-UTR, thereby decreasing the stability of its mRNA.

Pdx1, a key pancreatic transcription factor, not only activates the expression of *Insulin* and *Nkx2.2* but also promotes the transcription of cell cycle-related genes such as *Ccnd1* and *Ccnd2*, thereby facilitating pancreatic cell proliferation (Baumel-Alterzon and Scott, 2022). In *rbm38* mutants, although the expression levels of *ccnd1, ccnd2a*, and *ccnd2b* showed no significant changes compared to wild-type embryos, they were relatively higher in the absence of Rbm38 (Supplementary Figure S5B).

### Rbm38 regulates pancreatic development via alternative splicing

**Figure 8:**
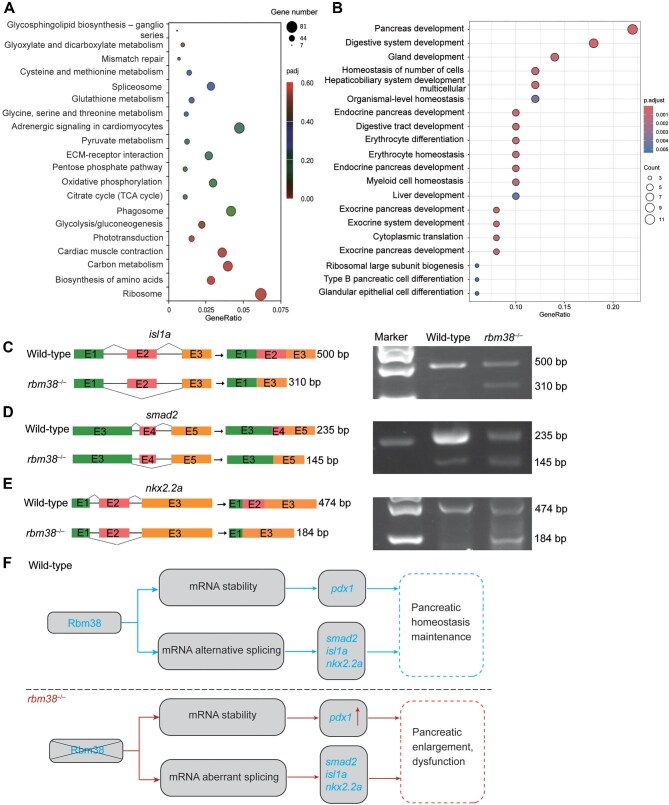
Rbm38-regulated pancreas-specific alternative splicing events. (**A**) KEGG pathway analysis of differentially expressed genes in *rbm38* mutants. (**B**) GO analysis shows enrichment of alternative splicing events in the pancreas. (**C**–**E**) Validation of altered splicing in *rbm38* mutants at 72 hpf. Boxes highlight exons that are differentially spliced between wild-type embryos and *rbm38* mutants. (**F**) Schematic diagram of the molecular mechanism underlying Rbm38 function in pancreatic development. The upper panel shows wild-type embryos, where Rbm38 maintains normal pancreatic development and tissue homeostasis by regulating the stability of *pdx1* mRNA and modulating the alternative splicing of *isl1a, nkx2.2a*, and *smad2*, thereby fine-tuning the expression of pancreas-related genes. The lower panel shows *rbm38* mutants, in which the loss of Rbm38 leads to increased stability of *pdx1* and dysregulated splicing of several key transcription factor genes, resulting in pancreatic enlargement.

To further explore the mechanism by which Rbm38 functions in pancreatic development, we performed RNA sequencing (RNA-seq) analyses of wild-type and *rbm38* mutant embryos at 72 hpf. Kyoto Encyclopedia of Genes and Genomes (KEGG) enrichment uncovered a significant number of differentially expressed genes in the glycolysis, gluconeogenesis, and citrate cycle pathways (Figure 8A). These pathways are integral to glucose metabolism and energy balance, both of which are critical for pancreatic function.

Rbm38 is a multifaceted RBP, regulating the stability of target mRNAs and also acting as a splicing factor. We employed rMATS to analyze the occurrence of five major types of alternative splicing events from RNA-seq data, including skipped exon, retained intron, mutually exclusive exon, alternative 5′ splice site, and alternative 3′ splice site. Given that the transcriptomic analysis was conducted on whole embryos at 72 hpf, we performed a Gene Ontology (GO) functional enrichment analysis on pancreas-specific genes (Figure 8B). The results revealed significantly altered alternative splicing events, suggesting that Rbm38 also plays a pivotal role in regulating the alternative splicing during pancreatic development.

We next experimentally validated the splicing defects of several key genes related to pancreatic development in *rbm38* mutants at 72 hpf. In line with the results from the rMATS analysis, there was a skipped second exon in a transcript variant of *isl1a* (Figure 8C), a skipped fourth exon in *smad2* (Figure 8D), and a skipped second exon in *nkx2.2a* (Figure 8E). These findings underscore the importance of Rbm38 in alternative splicing that is crucial for pancreatic development.

In summary, Rbm38 plays a crucial role in maintaining pancreatic homeostasis by destabilizing *pdx1* mRNA and regulating the alternative splicing of key transcription factors, such as *isl1a, nkx2.2a*, and *smad2*, thereby fine-tuning the expression of genes involved in pancreatic development. In the absence of Rbm38, the stability of *pdx1* mRNA is increased, and the splicing of several critical transcription factors is disrupted, leading to pancreatic enlargement and dysfunction (Figure 8F).

## Discussion

Pancreatic development is a complex process that relies on the coordinated regulation of gene expression at both transcriptional and post-transcriptional levels (Apelqvist et al., 1999; Hebrok, 2003; Fujitani et al., 2006; Glauser and Schlegel, 2009; Leung, 2010; Jennings et al., 2015; Mi et al., 2024). Recently, an increasing number of RBPs have been demonstrated to play critical roles in pancreatic development through post-transcriptional regulation. In this study, we found that Rbm38 is predominantly expressed in the pancreas during zebrafish development and contributes to pancreatic development by regulating alternative splicing and mRNA stability of pancreatic genes. The loss of its function results in abnormal pancreatic enlargement.

Rbm38 displays highly restricted yet diverse expression patterns in early embryos, highlighting its involvement in a range of developmental processes. Furthermore, *rbm38* exhibits dynamic expression within various tissues, including mesoderm, eyes, brain, spinal cord, neural plate, duodenum, liver, and hematopoietic tissues, as shown in *Xenopus* (Boy et al., 2004). Importantly, our WISH analyses, knock-in studies, and promoter reporter assays have uncovered that Rbm38 is highly expressed in the pancreas. This finding underscores the potential for Rbm38 in regulating pancreatic development. Indeed, we found that the loss of Rbm38 leads to abnormal enlargement of the pancreas during zebrafish development, accompanied by the upregulation of marker genes associated with both endocrine and exocrine glands. It has been shown that Rbm38-deficient mice exhibit symptoms such as hematopoietic disorders, splenomegaly, and cardiac enlargement (van den Hoogenhof et al., 2017). Furthermore, Rbm38 deficiency accelerates aging in mice and predisposes them to spontaneous tumor formation (Zhang et al., 2014). Although there is no definitive conclusion on whether the loss of Rbm38 affects pancreatic development in mice, the currently distinct phenotypes observed in zebrafish and mice implies that Rbm38 plays diversified roles in a tissue-specific and stage-specific manner.

RBPs typically associate with proteins and RNA to form RNP granules, which are involved in various cellular processes such as RNA transport, translation, and degradation (Pereira et al., 2017; Corbet and Parker, 2019). This is consistent with our observation that Rbm38 exhibits a granule-like distribution in SW1990 cells. Previous studies have shown that Rbm38 typically binds to AU-rich or GU-rich regions, which can regulate the degradation rate of mRNAs, thus affecting their half-life (Xu et al., 2013; Qian et al., 2020). We found that Rbm38 can recognize the CAGAUU sequence in the 3′-UTR of *pdx1* mRNA, thereby decreasing its stability. The transcription factor PDX1 is the earliest expressed protein during pancreatic development, playing a crucial role in early pancreas formation and maintaining the function of mature β-cells (Jonsson et al., 1994; Gao et al., 2014). In zebrafish and mice, *pdx1* exhibits highly similar expression patterns and functions, with predominant expression in pancreatic progenitor cells and mature β-cells. The loss of *pdx1* in zebrafish severely disrupts normal pancreatic development, leading to a significant decrease in the β-cell number and insulin secretion, resulting in the increased blood glucose levels and metabolic disorders (Kimmel et al., 2015; Bastidas-Ponce et al., 2017). In early pancreatic progenitors, Pdx1 is essential for driving proliferation and pancreatic growth, while at later stages, it plays a crucial role in lineage specification, proliferation, and maintenance of selected cell types (Babu et al., 2007). Elevated Pdx1 expression stimulates pancreatic β-cell proliferation and enhances insulin secretion (Regue et al., 2021). This is consistent with our findings in *rbm38* mutants, where increased *pdx1* mRNA stability leads to upregulated *insulin* expression and a concomitant decrease in blood glucose levels. The loss of Rbm38 results in the upregulation of Pdx1 expression level, which may partially account for the abnormal enlargement of the pancreas.

Accumulating evidence suggests that Rbm38 participates in various biological processes through multiple post-transcriptional mechanisms (Shu et al., 2006; Zhang et al., 2010; Lucchesi et al., 2019, 2023; Li et al., 2020). Previous studies suggested an essential role of Rbm38 in alternative splicing during late erythroid differentiation, neurogenesis, and myogenesis (Anyanful et al., 2004; Heinicke et al., 2013; Amrane et al., 2014; Ganaie et al., 2018; Li et al., 2020; Han et al., 2022). Consistently, we found that several important pancreas transcription factor genes, including *isl1a, nkx2.2a*, and *smad2*, were aberrantly spliced in Rbm38-deficient pancreas. The LIM-homeodomain transcription factor Isl1 plays a critical role in regulating the expression of numerous genes, including *Pdx1, Pax6*, and *Mafb*, thereby influencing pancreas morphology and endocrine cell differentiation (Du et al., 2009; Ediger et al., 2014; Bastidas-Ponce et al., 2017; Scavuzzo et al., 2018; Bakhti et al., 2019; Bohuslavova et al., 2023). In vertebrates, Isl1 exhibits a highly conserved expression pattern and is continuously expressed in all endocrine cells. In *isl1* mutant zebrafish, the initial specification of endocrine cells occurs normally, but more than half of these cells fail to successfully initiate endocrine hormone expression (Wilfinger et al., 2013). Interestingly, the *Isl1* gene has two splicing variants, *Isl1α* and *Isl1β*, whose abundance differs between tissues (Ando et al., 2003; Bejarano-Escobar et al., 2015). Specifically, *Isl1β* is the predominant form in the pancreas (Ando et al., 2003; Lin et al., 2013; Jung et al., 2018). In addition, *in vitro* studies have shown that Isl1 directly regulates *cyclin D1*, suggesting a potential link between Isl1 and islet cell proliferation (Liu et al., 2012). Nkx2.2 governs crucial cell fate decisions in several developing organs, including the central nervous system, pancreas, and intestine (Seymour and Serup, 2023). Smad2 is an intracellular signaling factor activated by TGF-β signals and plays a vital role in regulating pancreatic islet function and maintaining β-cell mass (Harmon et al., 2004; Nomura et al., 2014). Pdx1 and Isl1a promote pancreatic cell proliferation by activating the expression of cell cycle-related genes such as* ccnd1*, while Smad2 indirectly influences this process through its involvement in the TGF-β signaling pathway (Harmon et al., 2004; Liu et al., 2012; Nomura et al., 2014). Whether these regulatory effects converge on a common signaling cascade remains to be elucidated through further investigation.

Our findings suggest that Rbm38 plays a pivotal role in pancreatic development by regulating alternative splicing and mRNA stability. Previous studies have demonstrated that Rbm38 is involved in regulating cell cycle and apoptosis through multiple post-transcriptional mechanisms, including pre-mRNA alternative splicing and mRNA localization, stability, polyadenylation, and translation (Zhang et al., 2010, 2011; Xu et al., 2013; Zhang et al., 2013; Lucchesi et al., 2016). Rbm38 is expressed in skeletal muscle cells and contributes to the differentiation process, at least in part, by post-transcriptionally regulating muscle gene expression. A similar function has been reported for its nematode ortholog, SUP-12 (Miyamoto et al., 2009; Grifone et al., 2020). Rbm24 is another homolog of the *Caenorhabditis elegans* SUP-12 (Miyamoto et al., 2009; Ray et al., 2009; Heinicke et al., 2013; Grifone et al., 2020). Both Rbm24 and Rbm38 display highly restricted yet diverse expression patterns during early development across various species (Fetka et al., 2000; Boy et al., 2004; Miller et al., 2008; Maragh et al., 2011, 2014; Poon et al., 2012; Grifone et al., 2014). Their expression patterns show remarkable similarity, which is consistent with their redundant functions (Miyamoto et al., 2009; Sun et al., 2016; van den Hoogenhof et al., 2017; Grifone et al., 2020). Interestingly, they also exhibit distinct expression profiles, indicating cell type-specific functions. For example, Rbm24 is essential for lens differentiation by regulating poly(A) tail length of *crystallin* mRNAs (Shao et al., 2020), whereas Rbm38 is crucial for differentiation of retinal neurons in *Xenopus laevis* (Boy et al., 2004). Therefore, Rbm38 and Rbm24 display both specific and redundant functions during vertebrate development.

RBPs also act in concert and/or mutually interact to post-transcriptionally regulate tissue-specific gene expression (Montanes-Agudo et al., 2023; Shi, 2024). Studies have demonstrated that Rbm38 collaborates with HuR to modulate the stability of *p21* mRNA (Cho et al., 2010), while Rbm24, Rbm38, and PTBP1 function collectively to control the alternative splicing of exon 68 in *Cdh23* (Li et al., 2020). Based on the String database, Rbm38 can interact with several other RBPs, many of which are important for pancreatic function (Bennett et al., 2010; Kuwasako et al., 2014; Moss and Sussel, 2020). For example, Rbfox1 and Rbfox2 are required for insulin secretion and blood glucose homeostasis (Juan-Mateu et al., 2017). Additionally, conditional mutation of *Rbfox2* in the mouse pancreas leads to reduced insulin secretion and compromised blood glucose regulation (Moss et al., 2023). Whether Rbm38 interacts with Rbfox1, Rbfox2, or other RBPs during pancreatic development awaits future investigation.

In conclusion, we identified a specific expression of zebrafish Rbm38 in the pancreas and a novel role of this protein in pancreatic development. Our study also revealed an essential role of Rbm38 in regulating pre-mRNA alternative splicing and mRNA stability of key pancreatic genes.

## Materials and methods

### Zebrafish, embryo, and cell culture

The zebrafish AB strain was raised at 28.5°C in the standard zebrafish culture system (Haisheng, China). The *Tg*(*fabp10a*:*dsRed;ela3l*:*GFP*) transgenic line was purchased from the China Zebrafish Resource Center. Embryos were maintained at 28.5°C in a temperature-controlled incubator. To inhibit pigment formation, embryos were cultured in medium containing 0.0045% 1-phenyl-2-thiourea starting from 24 hpf.

SW1990 and HEK293T cells were cultured in Dulbecco's modified Eagle medium (DMEM) supplemented with 10% (*v*/*v*) fetal bovine serum (FBS; YEASEN) at 37°C in a humidified incubator with 5% CO_2_.

This study was performed according to the standard animal guidelines and approved by the Animal Care Committee of Ocean University of China (OUC-AE-2018108).

### Expression profile analysis and transcription factor-binding site prediction

The expression profile of zebrafish *rbm38* was retrieved from online single-cell transcriptome database (https://daniocell.nichd.nih.gov/index.html). Putative transcription factors binding to the *rbm38* promoter were identified via AnimalTFDB 4.0 (https://guolab.wchscu.cn/AnimalTFDB4/). Potential Rbm38-binding sites on *pdx1* mRNA were predicted using RBPmap (https://rbpmap.technion.ac.il/) and RegRNA 2.0 (http://regrna2.mbc.nctu.edu.tw/).

### WISH and FISH

WISH experiments were conducted according to the standard protocol (Thisse and Thisse, 2008). Digoxigenin-labeled anti-sense probes were *in vitro* synthesized using SP6 RNA polymerase (Thermo Fisher) or T7 RNA polymerase (Promega). Stained embryos were imaged using a stereomicroscope (Nikon, SMZ1000). FISH experiments were performed by following the standard protocol (He et al., 2020), and images were acquired using confocal microscopy (Andor Dragonfly 600). The fluorescence area was quantified by ImageJ.

### RT-qPCR

Embryonic tissues were homogenized in TRIzol, and total RNAs were extracted using the Total RNA Kit I (Omega). The cDNAs were synthesized using M-MLV reverse transcriptase (TaKaRa) in the presence of random primers following the manufacturer's instructions. RT-qPCR was performed to examine the expression profiles of *rbm38* at the various development stages using *gapdh* as an input control.

### Analysis of Rbm38 subcellular localization

The pcDNA3.1/V5/GFP vector was generated by inserting the *GFP* gene into the eukaryotic expression vector pcDNA3.1/V5-His A. The amplified *rbm38* open reading frame was cloned into the pcDNA3.1/V5/GFP vector. Plasmids were transfected into SW1990 cells using the HieffTrans™ Liposomal Transfection Reagent (YEASEN) according to the manufacturer's instructions. Transfected cells were cultured in DMEM supplemented with 5% (*v*/*v*) FBS without penicillin and streptomycin for 24 h and then stained with DAPI, followed by washes with phosphate-buffered saline (PBS). Fluorescent images were acquired using a confocal microscope (Leica Sp8).

### Generation of zebrafish rbm38 mutant and rbm38-GFP knock-in lines

The zebrafish *rbm38* mutant line was generated using the CRISPR/Cas9 method by targeting the first exon. The sgRNA (5′-GGGCGACATCGATGAAGCGG-3′) was synthesized by *in vitro* transcription, and 200 pg of this sgRNA along with 320 pg of Cas9 protein (NEB) were injected into zebrafish embryos at the 1-cell stage. At 24 hpf, pooled genomic DNAs from 6–8 embryos were extracted using 25 μl of NaOH (50 mM), followed by neutralization with 25 μl of Tris–HCl (40 mM). The DNAs were then amplified using specific genotyping primers (Supplementary Table S1), and the resulting PCR products were subjected to Sanger sequencing. Upon confirmation of successful targeting, the remaining embryos were raised to adulthood and screened for the mutation. The *rbm38-GFP* knock-in zebrafish line was generated by following the established protocol (Levic et al., 2021), with some modifications. Specifically, the primers used for template amplification were modified with a phosphorothioate bond at the 5′ end of the first five bases. This modification has been shown to protect the template from degradation by exonuclease activity (Zhang et al., 2023). An sgRNA targeting the last intron of *rbm38* (5′-TGAGGACTCGCAAAGTA-3′) was synthesized by *in vitro* transcription.

### Immunofluorescence and pancreas size quantification

Zebrafish embryos at 72 hpf and 96 hpf were fixed using 4% paraformaldehyde at 4°C overnight. Immunofluorescence staining was performed according to the standard protocol (Li et al., 2025). The following antibodies were used: rabbit anti‐carboxypeptidase A (1:400, MCE, YA2486), mouse anti-GFP (1:400, Roche, 11814460001), Alexa Fluor™ 488-conjugated goat anti-mouse IgG (H+L) cross-adsorbed secondary antibody (1:400, Invitrogen, A-11001), and Alexa Fluor™ 555-conjugated goat anti-rabbit IgG (H+L) cross-adsorbed secondary antibody (1:400, Invitrogen, A-21428). Nuclei were counterstained with DAPI, and embryos were imaged using a confocal microscopy (Andor, Dragonfly 600).

Embryos derived from crossing between *rbm38* mutants and the transgenic line *Tg*(*fabp10a*:*dsRed;ela3l*:*GFP*) were imaged using a fluorescence microscope (Leica, M205 FA). The fluorescence area was quantified by ImageJ.

### Glucose quantification

Glucose quantification experiments were conducted according to the standard protocol (Sancho et al., 2010), with some modifications. Zebrafish embryos (30 per group) were collected at 72 hpf and 96 hpf, respectively. After washing three times with PBS, embryos were homogenized in 50 μl of PBS. Glucose levels were then quantified using a Glucose (GOD-POD) Assay Kit (Yeasen, 60408ES60) following the manufacturer's instructions.

### Analysis of mRNA stability

The mRNA stability was analyzed by following a standard protocol with minor modifications (Zhang et al., 2017). Zebrafish embryos (30 for each condition) at 72 hpf were treated with 20 μg/ml of actinomycin D (MCE) or DMSO for 4 h and 8 h. HEK293T cells were treated with 10 μg/ml of actinomycin D or DMSO for 2 h, 4 h, 6 h, and 8 h. After treatment, total RNAs were extracted from the tails of zebrafish embryos or HEK293T cells for reverse transcription and RT-qPCR analysis.

### Luciferase assays

HEK293T cells were transfected with plasmids using the HieffTrans™ Liposomal Transfection Reagent. After 24 h, cells were lysed, and the activity of firefly luciferase and renilla luciferase was measured using the Dual-Luciferase Reporter Assay System according to the manufacturer's instructions (YEASEN). Renilla luciferase was used as an internal control.

### Statistical analysis

Quantitative data were expressed as mean ± standard deviation (SD) from at least three independent experiments. Statistical analysis was analyzed by one-way analysis of variance (ANOVA) using the software GraphPad Prism. *P* < 0.05 was considered as statistically significant.

## Supplementary Material

mjaf025_Supplemental_File

## Notes

**Author contributions:** H.L., S.Y., and X.Z. designed the research; X.Z. and X.L. performed the experiments; L.W. analyzed RNA-seq data; S.Y., R.L., X.Z., A.Z., and Y.W. analyzed the data; X.Z. and H.L. wrote the manuscript. All authors contributed to the article and approved the submitted version.
