# Supplementary material for: RNA-binding protein Rbm38 as a multifaceted post-transcriptional regulator in zebrafish pancreatic development
Source: J Mol Cell Biol. 2025 Aug 12;17(6):mjaf025. doi: 10.1093/jmcb/mjaf025 (PMC12866639; doi:10.1093/jmcb/mjaf025)
Supplement: mjaf025_Supplemental_File [file mjaf025_supplemental_file.pdf]

RNA-Binding Protein Rbm38 as a Multifaceted Post-Transcriptional Regulator in Zebrafish Pancreatic Development

Xiangmin Zhang[1], Xianpeng Li[1], Rui Liu[1], Lu Wang[1], Yunchao Wang[1], Ailong Zhang[1], Shuaiqi Yang[1*] and Hongyan Li[1,2*]

[1]College of Marine Life Sciences, Key Laboratory of Evolution & Marine Biodiversity (Ministry of Education) and Institute of Evolution & Marine Biodiversity, Ocean University of China, Qingdao 266003, China

[2]Laboratory for Marine Biology and Biotechnology, Qingdao Marine Science and Technology Center, Qingdao 266003, China.

[*]Corresponding authors

Dr. Hongyan Li

Room 301, Darwin Building, 5 Yushan Road, Ocean University of China, Qingdao 266003, China

Tel: +86 532 82032092

E-mail: hongyanli@ouc.edu.cn

Dr. Shuaiqi Yang

Room 213, Darwin Building, 5 Yushan Road, Ocean University of China, Qingdao 266003, China

Tel.: +86 532 82032092

E-mail: ysq@ouc.edu.cn

Supplementary Figure S1

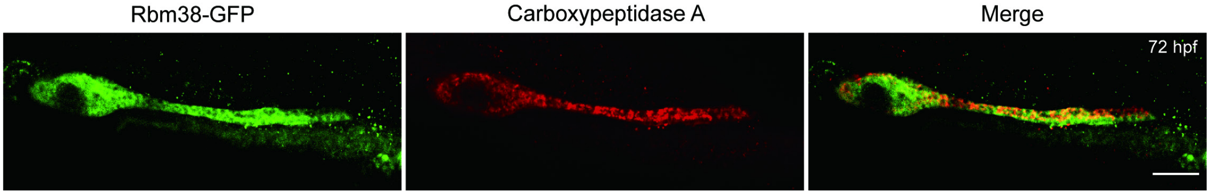

Supplementary Figure S1. Rbm38 is specifically expressed in the zebrafish pancreas. Immunofluorescence staining showing co-localization of Carboxypeptidase A and GFP signals in *rbm38-GFP* knock-in embryos at 72 hpf. Scale bars, 50 µm.

Supplementary Figure S2

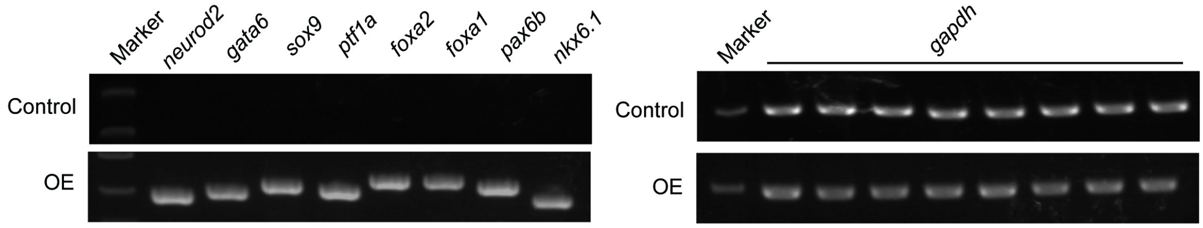

Supplementary Figure S2. Expression of pancreatic transcription factors after transfection in HEK293T cells. RT-PCR analysis confirms the overexpression (OE) of each transcription factor in the HEK293T cell line, using pcDNA3.1 empty vector as a control and *gapdh* as an internal reference.

Supplementary Figure S3

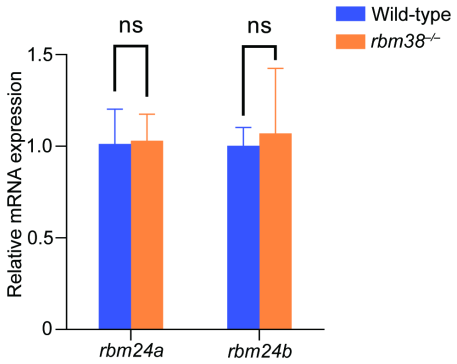

Supplementary Figure S3. Expression of *rbm24a* and *rbm24b* in *rbm38* mutants. The expression levels of *rbm24a* and *rbm24b* show no significant differences between wild-type embryos and *rbm38* mutants at 72 hpf.

Supplementary Figure S4

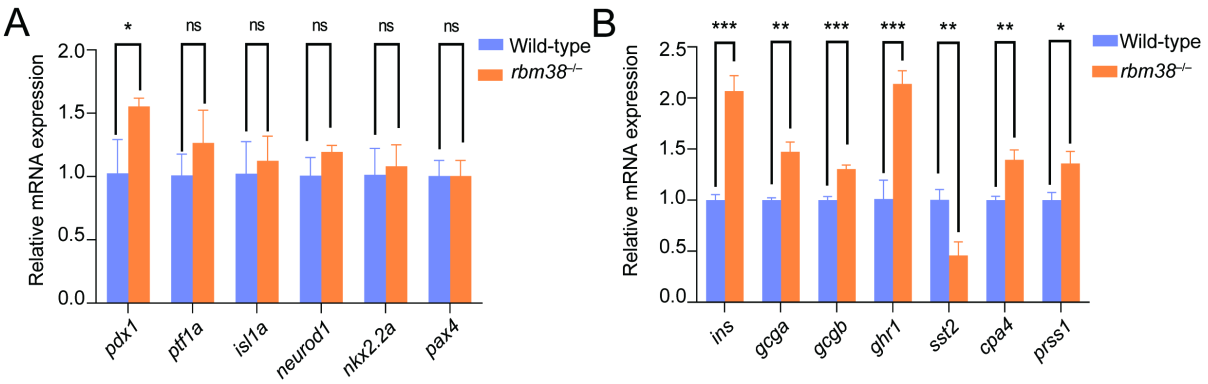

Supplementary Figure S4. Loss of Rbm38 disrupts the expression of pancreatic marker genes. (A) Relative expression levels of key pancreatic transcription factors in wild-type embryos and *rbm38* mutants at 96 hpf, normalized to *gapdh*, with values in wild-type embryos set to 1. Data are presented as mean ± SD, and analyzed by Student's *t*-test (*, $p < 0.05$; ns, not significant). (B) Quantitative analysis of the expression of endocrine and exocrine marker genes in wild-type embryos and *rbm38* mutants at 96 hpf, normalized to *gapdh*, with values in wild-type embryos set to 1. Data are presented as mean ± SD, and analyzed by Student's *t*-test (*, $p < 0.05$; **, $p < 0.01$; ***, $p < 0.001$; ns, not significant).

Supplementary Figure S5

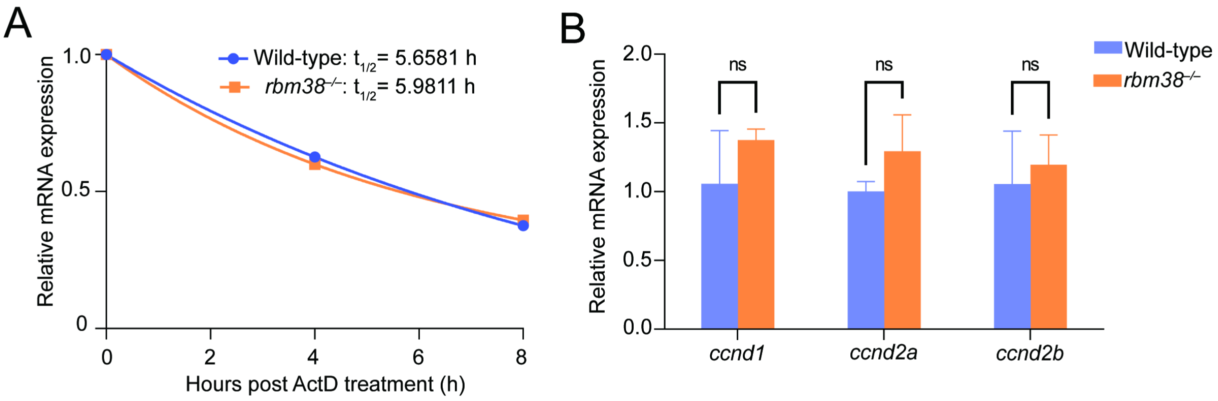

Supplementary Figure S5. Rbm38 does not affect the stability of *ptf1a* mRNA. (A) The stability of *ptf1a* mRNA was analyzed in wild-type embryos and *rbm38* mutants at 72 hpf after actinomycin D treatment. (B) Relative expression levels of *ccnd1*, *ccnd2a* and *ccnd2b* in wild-type embryos and *rbm38* mutants at 72 hpf, normalized to *gapdh*, with values in wild-type embryos set to 1. Data are presented as mean ± SD, and analyzed by Student's *t*-test (ns, not significant).

Supplementary Table S1

Supplementary Table S1. Primers utilized in this study

**Name of Primer (RT-qPCR)**	**Sequence (5′ to 3′ Direction)**
rbm38 F	GCACAGATGCTTCTACATCAGTTCGT
rbm38 R	GTCCGTTATCACCACCGCTTCAT
pdx1 F	CATTACTATCCGCCTAACCACCTGTA
pdx1 R	GCGACTGTAGAGATGTCTGTGAAGT
ptf1a F	AGACCACTTGGACACAGACGACTT
ptf1a R	TCCGCCTCTTCAGTAAGCCTCC
insulin F	CTGTTGGTCCTGTTGGTCGTGTC
insulin R	CTGCTCTACAATGCCTCTCTTCCTTATC
nkx2.2a F	GCCGTGCCAGTGTTAGTCAGAG
nkx2.2a R	GCAAGTCAGTATTTGTTCACCAAGTCC
isl1a F	TTCATGTGTTCGTGTCCGTCCAA
isl1a R	TGGCGTTGTTCTTCCATCAGTCTT
pax4 F	ACTCCTGAAGTCATCTCCATCATAGTTC
pax4 R	TGCTGTGAATGCGGTTCTGCTT
neurod1 F	GCCACACTAGACTTCAATCCGTAGG
neurod1 R	CGGCATCATCGTCGTCTTCCATAT
gcga F	CGACAGCACAAGCACAGAGACAG
gcga R	CATGACGTTTGACAGAACCACCATTTC
gcgb F	CGTGCTATTTAATGACCCAAGAGAAGT
gcgb R	ATCACTGGTGTATGTGCCGTCTG
sst2 F	CTCCTCGCAACTCCACCTGACA
sst2 R	TTCTCATAAGCTCCAGTCCACTGTGTA
ghrl F	CCTGTGTGTTTCTCTTTCCTTGTGTCT
ghrl R	AGCACGGGACCATATTTCTCATATTCAG
prss1 F	ACACCACTCAACAGCTTCACCATG
prss1 R	GACTTGTAGCAGTGAGCAGCAGAC
ccnd1 F	ACACTTCCTTGCCAAACTGCCTAT
ccnd1 R	TCACTTCTGATGACTTGCGAGAGG
ccnd2a F	TGGATGTTAGAGGTCTGCGAGGAA
ccnd2a R	CAGTTCTTGCGGTCTGATTGAGTTATC
ccnd2b F	AGGTGTGTGAAGAGGAGAAATGTGAAG
ccnd2b R	GCAGTTGTTGAGATGTAATGGAGTTGTC
neurod2 F	GAGGAAGACTATGACGAGGATGTTGATG
neurod2 R	TAGCACGGTACGACCTTGAGTAGATT
foxa1 F	CCCTATTCCTACATCTCCCTCATCACA
foxa1 R	CTTCGCAGGTAACAGCCATTCTCAA
foxa2 F	CTCTATCCGCCACTCGCTGTCT
foxa2 R	TCGTTACTTGAAGAGTTGGAATGAGGAG

gata6 F	TGCTGGAGGAGATGGTGGAGAG
gata6 R	AGTGTTGTGGTGCTCGTCTGAC
pax6b F	GGAGGAGAGAGGAGAAACTGAGGAA
pax6b R	GGAATAGGAAGATGTCTGGCTTGGTT
sox9 F	TGAAGATGGAGAGCAGACGCAGAT
sox9 R	GTCCACCGCACCGAAGTCAATG
nkx6.1 F	CCGCAGGAATCTTGTCTGGACTG
nkx6.1 R	AGCACGCAAATCTGGCATCTCTC
ptf1a F	AGACCACTTGGACACAGACGACTT
ptf1a R	TCCGCCTCTTCAGTAAGCCTCC
rbm24a F	CGACGGCAGGAAGGCTAATGTTAA
rbm24a R	AGGTTGCTGTTGGCTGGATGTG
rbm24b F	ACACATCAACTCGCTCGTTCACTT
rbm24b R	TGCCAGTCTGCCTGTCAGTAATAAC
**Name of Primer (WISH)**	**Sequence (5′ to 3′ Direction)**
rbm38 F	ACAGACAGAGGAGGAGGAGTGC
rbm38 R	CTGTCCCACAATGACCGTGTTCTA
prss1 F	CCACTCAACAGCTTCACCATGA
prss1 R	ACACGCCATGATAACGACCTCAA
insulin F	CCATATCCACCATTCCTCGCCTCT
insulin R	TCATTTGATAGTTTTATTTTTTCTGTAAAAACAAACGGAGAG
**Name of Primer (AS)**	**Sequence (5′ to 3′ Direction)**
isl1a F	CCTAACTCTAACGCAAGCATCTCGATT
isl1a R	CTGGGATGAGCTGCCGACTACA
nkx2.2a F	GGAGATATTTCATTAACTGTGCGGTGTC
nkx2.2a R	CTTACCAGAGTCGCTGCCGTTG
smad2 F	CAACTGGACGAGCTGGAGAAGG
smad2 R	CAGGTCGGGCCATCGCCACAGGCGG
**Name of Primer (CDS)**	**Sequence (5′ to 3′ Direction)**
pax6b F	ATGCCTCAAAAAGAATACCATAACC
pax6b R	TTACTGTAATCTTGACCAGTATTGAGACA
sox9b F	ATGAATCTCCTCCAGCGCG
sox9b R	TCAGGGTCTGGACAGCTGTGT
gata6a F	ATGGACCTGGGTGATAACAGCT
gata6a R	TTAGGCCAAGGCCATGGG
foxa2 F	ATGCTCGGTGCTGTCAAAATG
foxa2 R	TTAGGAAGAGTTCAGGATGGGC
foxa1 F	ATGTTGGGCGCAGTGAAAAT
foxa1 R	TACGATGTATTGAGAACAGGCCTG

nkx6.1 F	ATGTTAGCGGTGGGGCAAA
nkx6.1 R	TTACGAGCTCTCGTTTTCCGA
neurd2 F	ATGTTAACAAGATTATTCAAAGAACCTTC
neurd2 R	CTAACTGTGGAAAAAGGCGTTGA
ptf1a F	ATGGACACTGTGTTGGATCCATTC
ptf1a R	TTAGGAAATGAAATTAAAGGGGGGT
rbm38 F	ATGCTTCTACATCAGTTCGTGAA
rbm38 R	CTAGAGGCGGTCGGTGTGGAGCTGG
**Name of Primer (KO)**	**Sequence (5′ to 3′ Direction)**
rbm38 sg	TAATACGACTCACTATAGGGCGACATCGATGAAGCGGGTTTTAGAGCTAGAAATAGC
rbm38 F	TTCAGTTGTTCACCTGCCGTCATT
rbm38 R	TTTACTCACAAAGCCGTATCCTCTGG
**Name of Primer (KI)**	**Sequence (5′ to 3′ Direction)**
rbm38 sg	taatacgactcactataGGGTGAGGACTCGCAAAGTAgttttagagctagaa
donor F	C*A*C*C*TTGTCAACTTTGCTCTTCTTTATTTGCCTCGTCT
donor R	A*A*G*C*T*GGCCTCAGCGAACACTCGTCTGTCCCTGGAGTGTCA
	* indicates a phosphorothioate bond
**Name of Primer (Trunc)**	**Sequence (5′ to 3′ Direction)**
rbm38△1 F	TAGGGAGAACTGAAACGCCAACTGAGCCGAGGT
rbm38△1 R	GCGTTTCAGTTCTCCCTACCTTCGCGTGGGTTT
rbm38△2 F	AGTCTTGCTGTGAGGCAACAGCACTACCTACTG
rbm38△2 R	TTGCCTCACAGCAAGACTCGGCTCAGTTGGCGTTT
rbm38△3 F	ATAGGTGCAAATATGGGCCAGTTATCTCAAGAC
rbm38△3 R	GCCCATATTTGCACCTATACTTTAGGTTTAGACCCAGCTAG
rbm38△4 F	ACAGAGGAGGAGAGCACAGCGTCTCTCAGCAGC
rbm38△4 R	TGTGCTCTCCTCCTCTGTCTGTGACGCGCTCTC
rbm38△5 F	CTCTCAGCAACTGGGTAATGAGCTGCAGCAGCG
rbm38△5 R	TTACCCAGTTGCTGAGAGACGCTGTGCTGTGCA
rbm38△6 F	GGCGCACTGGCTGCAGCAGCGCGCGGTCATTCA
rbm38△6 R	TGCTGCAGCCAGTGCGCCGCCGCCGCTGCTGAG
pdx1△1 F	AACTCCAACAGTGCTAGCAAACAGATTGGCAAA
pdx1△1 R	GCTAGCACTGTTGGAGTTTTACAACTGCTGTCAGTC
pdx1△2 F	GTACAGCAAACAGATTGGCAAAAGGGGGCGCTC
pdx1△2 R	CCAATCTGTTTGCTGTACATATTGGAGTTTTACAACTGCTG
pdx1△3 F	TACAGTGCTCAGATTGGCAAAAGGGGGCGCTCT
pdx1△3 R	GCCAATCTGAGCACTGTACATATTGGAGTTTTACAAC
